# Editorial

**DOI:** 10.1120/jacmp.v2i3.2602

**Published:** 2001-09-01

At the recent annual meeting of the American College of Medical Physics the annual awards for the best papers published in the JACMP during the previous year were handed out. The papers were selected by the Awards and Honors Committee of the American College of Medical Physics.

The LAP Award for Excellence for the best radiation oncology physics paper went to Michael G. Herman, Jon J. Kruse, and Christopher R. Hagness for their paper, “Guide to Clinical Use of Electronic Portal Imaging.”

The RIT Award for Excellence for the best diagnostic imaging paper went to Benjamin R. Archer and Louis K. Wagner for their paper, “Protecting Patients by Training Physicians in Fluoroscopic Radiation Management.”

The Elekta Award for Excellence for the best professional paper went to Peter Dunscombe, Gisele Roberts, and Lianne Valiquette for their paper, “Preventive Maintenance and Unscheduled Downtime from an Economic Perspective.

Each paper received an award of $500 and each author received a framed certificate.

Congratulations to all the winners for the excellent papers and our thanks and appreciation to LAP, RIT, and Elekta for making it possible.

The question has been raised about the right of authors to post their manuscripts on their own Web pages. Authors may do so within specific guidelines, regarding copyright, article citation, and restriction to personal use. Complete information can be found at the journal Web page under Information to Authors, paragraph 10.

Peter R. Almond, Ph.D.

Editor‐in‐Chief

